# RNAi by Soaking *Aedes aegypti* Pupae in dsRNA

**DOI:** 10.3390/insects12070634

**Published:** 2021-07-13

**Authors:** Fiza Arshad, Arvind Sharma, Charleen Lu, Monika Gulia-Nuss

**Affiliations:** Department of Biochemistry and Molecular Biology, University of Nevada, Reno, NV 89557, USA; farshad75@nevada.unr.edu (F.A.); arvinds@unr.edu (A.S.); charllu@nevada.unr.edu (C.L.)

**Keywords:** *Aedes aegypti*, RNA-interference, gene-silencing, pupal RNAi

## Abstract

**Simple Summary:**

Even after decades of control interventions, mosquito-borne diseases still pose a huge threat to humans. Understanding gene functions is important for discovering new targets for mosquito and mosquito-borne disease control. One of the well-established and widely used methods for understanding gene function is RNA-interference (RNAi). The use of RNAi is, however, restricted mostly to adult mosquitoes. A few studies have shown its applicability in mosquito larvae, and just one in pupae. The current double-stranded RNA (dsRNA) delivery methods for RNAi are labor-intensive and require microinjections into mosquitoes (adults, larvae, or pupae). In this study, we present a simple, fast, and less labor-intensive technique for RNAi in the pupal stage by soaking pupae in water containing dsRNA. This method will be useful in studying genes expressed in immature life stages of the mosquitoes and will hopefully open new avenues for identifying mosquito control targets in early life stages.

**Abstract:**

RNA-interference (RNAi) is a standard technique for functional genomics in adult mosquitoes. However, RNAi in immature, aquatic mosquito stages has been challenging. Several studies have shown successful larval RNAi, usually in combination with a carrier molecule. Except for one study in malaria mosquito, *Anopheles gambiae*, none of the previous studies has explored RNAi in mosquito pupae. Even in the study that used RNAi in pupae, double stranded RNA (dsRNA) was introduced by microinjection. Here, we describe a successful method by soaking pupae in water containing dsRNA without any carrier or osmotic challenge. The knockdown persisted into adulthood. We expect that this simple procedure will be useful in the functional analysis of genes that highly express in pupae or newly emerged adults.

## 1. Introduction

The introduction of exogenous double-stranded RNA (dsRNA) into the cells of diverse eukaryotic organisms, the mechanism known as RNA interference (RNAi), has been shown to induce rapid and sustained degradation of mRNAs containing sequences complementary to the dsRNA [1]. Because of its relative ease, RNAi has become the powerful and most widely used reverse genetics research tool in invertebrates for examining gene function [2]. It also holds great potential to contribute to novel strategies for species-specific control of insects.

The most common method for delivering the dsRNA in insects is by injection [3,4,5,6,7,8,9]. In mosquitoes, adult injections have been most successful. While microinjection in mosquito larvae [10,11,12] and pupae [13] has been shown to work, it is time-consuming and has little field application. An alternative to the microinjection technique is an uptake of dsRNA through ingestion (reviewed in [14]). In *Aedes aegypti,* moderate knockdown (30–50%) of the target gene was achieved by simply soaking larvae in a solution of dsRNA for 2 h [15]. In *Culex pipiens* larvae, effective dsRNA delivery and gene knockdown (77%) was demonstrated by dehydrating larvae in a salt solution followed by rehydration in water containing dsRNA, and the gene-silencing effect persisted through the pupal and adult mosquitoes [16]. In *Anopheles stephensi* mosquito larvae, microalgae were used as the vectors for carrying dsRNA [17]. Another alternative involves feeding mosquito larvae dsRNA that is incorporated into chitosan nanoparticles coated with agarose gel [18]. Another feeding protocol—embedding dsRNA into an edible effectene transfection buffer and feeding mosquito larvae for 16 h—was used to knock down a target gene in *Ae. aegypti* larvae [19]. While these techniques have great potential, it necessitates the creation of chitosan/dsRNA nanoparticles, and the creation of edible material of the correct size and dsRNA content to knock down the expression of target genes. Neither technique has been shown to have prolonged knockdown effects that persist into adulthood. It is apparent from the above summary that most RNAi protocols have focused on adults or larvae and the pupal RNAi has not yet gained attention. Here we present a simple technique for administering dsRNA to mosquito pupae that is quick, straightforward, easily repeatable, and has an effect that persists through the pupal stage and into adulthood.

## 2. Materials and Methods

Mosquito rearing: *Ae. aegypti* (UGAL strain) colonies were maintained at 27 °C and 78% relative humidity (RH) with a photoperiod of 16 h light and 8 h dark in a dedicated insectary. Eggs were hatched overnight in small plastic cups in deionized water. First instar larvae were counted (150 per pan) and reared in 500 mL of deionized water on a powdered fish food diet (Tetramin^®^, Melle, Germany), as described in detail in Pooraiiouby et al. [20]. Newly molted pupae (molted within 4 h) were used.

Quantitative expression profile: CYP4G35 (NCBI reference number: XM_001658018; also referred as CYP4g15) expression in different life stages: eggs, second and fourth instar larvae, early and late pupae, sugar- and blood-fed females, and sugar-fed males was carried out with qRT-PCR. Total RNA was isolated from a pool of samples per replicate (three replicates per cohort) using TRIzol reagent according to manufacturer’s protocols (Invitrogen, Waltham, MA, USA). Total RNA quantity was measured with a Nanodrop spectrophotometer. Five µg of total RNA was used for DNase treatment (Sigma, St. Louis, MO, USA) according to the manufacturer’s protocol. DNase-treated RNA samples were re-purified with TRIzol. Purity was determined by 260/280 and 260/230 ratios, with acceptable values in the ~2.0 and 2.0–2.2 range, respectively (all samples collected met purity standards). For cDNA synthesis, 1 µg of DNase treated RNA was used, with iScript reverse transcription supermix (BioRad, Hercules, CA, USA). cDNA was diluted 10× in RNase/DNase free water before using it as a template in qRT-PCR experiments. In each 10 µL qRT-PCR reaction, 1 µL cDNA was used. Each sample was run in triplicate wells of a 96-well plate. qRT-PCR was performed on a CFX touch Real-Time PCR Detection System (BioRad, Hercules, CA, USA), using SYBR green master mix (BioRad) and CYP4G35 specific primers: CYP4G35 Fwd 5′ATGCCAATATGCTGCTGCTGGGG 3′ and CYP4G35 Rev, 5′ CTGGCTCGGTCAAAAACACC 3′. Primers were also designed for a ribosomal protein S7 gene: S7 Fwd 5′ACCGCCGTCTACGATGCCA3′ and S7 Rev, 5′ATGGTGGTCTGCTGGTTCTT 3′ which was used as a housekeeping control. All reactions were performed with 3 min at 95 °C, followed by 39 cycles of 10 s at 95 °C, 30 s at 58 °C, and 30 s at 72 °C, and melt curve was analyzed at 65–95 °C. Relative expression was calculated using the 2^−ΔΔCt^ method, where eggs were considered control and expression of CYP4G35 in other life stages was calculated relative to the control levels. Experiments were replicated with three different mosquito cohorts.

dsRNA Preparation: Template for dsRNA synthesis was amplified from head cDNA using CYP4G35 primers designed with T7 promoter sequence T7AaCYP4G35 Fwd 5′TAATACGACTCACTATAGGGAGATGCCGATCTGACCGATGAAG3′ and T7AaCYP4G35 Rev 5′ TAATACGACTCACTATAGGGAGA TCTGTGTGCGTTCCGGTAG 3′. PCR conditions were 95 °C for 5 min, 95 °C for 30 s 55 °C for 30 s, 72 °C for 30 s (5 cycles) followed by 95 °C for 30 s, 65 °C for 30 s, 72 °C for 30 s (35 cycles) and a final extension at 72 °C for 10 min. The PCR product was purified (Zymo PCR purification kit) and 1 µg purified product was used for in vitro transcription. EGFP transcript was amplified from an EGFP plasmid (a gift from Prof. Michael Riehle, University of Arizona). EGFP primers were also designed with T7 promoter sequence T7EGFP_Fwd 5′TAATACGACTCACTATAGGGCCTGGTCGAGCTGGACGGCGAC3′ and T7 EGFP_Rev 5′TAATACGACTCACTATAGGGTCACGAACTCCAGCAGGACCAT3′. CYP4G35 (425 bp) and EGFP dsRNA (631 bp) were prepared following the manufacturer’s protocol (Ambion Megascript T7 in vitro transcription kit, Austin, TX, USA) and as previously described [7,21]. The high concentrations of dsRNA required for soaking were achieved by scaling up the in vitro transcription reaction. We used 1 µg RNA in a 20 µL reaction and a total of 5 reactions per gene. We also increased the incubation time to 16 h. With these modifications, we were able to achieve 25 µg dsRNA/µL. DsRNA was diluted and added to 500 µL of nuclease-free deionized (DI) water in the 12-well plate at a concentration of 5 µg/500 µL DI water (2 µL dsRNA at a concentration of 2.5 µg/µL).

RNAi set up: Our strict feeding regimen resulted in synchronized pupation where male pupae started to appear 10–12 h before females. We set up 6–8 trays of 150 larvae each. All the pupae were removed from trays in the morning and newly molted pupae were collected within 4 h, resulting in 0–4 h old pupae that were still light brown. To avoid any damage to the newly molted, fragile pupae, we used a paintbrush to remove them from trays and transferred to the 12-well plate (Corning™ Costar™ Flat Bottom Cell Culture plates, Corning, New York, NY, USA). Five pupae were kept in each well.

An amount of 500 µL of nuclease-free DI water was added to each well containing five pupae. Once pupae acclimatized to the wells (10–15 min), any dead pupae within this time were removed and replaced with new individuals. 5 µg (2 µL) dsRNA was addedto each well. We calculated dsRNA concentration based on our previous successful adult injections where we injected 1–2 µg dsRNA in 0.5 µL solution [7]. An EGFP dsRNA was used at an equal concentration as a control.

For pool assay, 30 pupae were treated with dsRNA in a small tissue culture dish (40.28 mm, Corning, New York, NY, USA). An amount of 30 µg (12 µL) of dsRNA was added to 4 mL of DNase-free DI water. Control plate contained 4 mL of nuclease-free DI water and 30 µg of dsEGFP or DI water only. Additional 1 mL of DI water was added on day 2 of treatment in the experimental and control plates.

Knockdown validation: Pupae were kept in cages for adult emergence. RNA was extracted from individual 2 d old adult mosquitoes. The iScript™ cDNA Synthesis Kit (BioRad, Hercules, CA, USA) was used to convert 500 ng–1 μg total RNA samples into cDNA following the manufacturer’s protocols. Briefly, cDNA was diluted 10× with DI H_2_O before using it as a template in qRT-PCR experiments. In each 10 μL reaction, 1 µL of cDNA was used. The reaction was set up as described above for the Expression Profile.

Statistical Analysis: Data were analyzed using GraphPad Prism 7 software (La Jolla, CA, USA). Relative expression values of CYP4G35 expression in different developmental stages and knockdown validation in three different groups (DI water, dsEGFP, and dsCYP4G35) were compared with one-way ANOVA, followed by Tukey multiple comparison test.

## 3. Results

Gene selection: Cytochromes P450 (CYP) enzymes constitute one of the largest gene family among all living organisms. CYPs are heme thiolate proteins that are known to catalyze xenobiotic metabolism and epoxidation. The insect CYPs can be divided into four major clans: CYP2, CYP3, CYP4, and mitochondrial.

The CYP4G subfamily is evolutionarily conserved across insects [22] but is absent in other orders such as Crustacea and Chelicerata. Insect genomes sequenced so far possess one or two CYP4Gs. Previous work in other insects and mosquito *A. gambiae* has shown that the CYP4G enzymes, in association with its reductase partner (Cytochrome P450 reductase, CPR), catalyze the last two steps in cuticular hydrocarbon biosynthesis and are therefore known for this alcohol oxidase and aldehyde decarbonylase activity [23]. Cuticular hydrocarbons are important for mosquitoes when transitioning from the aquatic to the terrestrial stage to avoid desiccation. *Ae. aegypti* genome has two CYP4G genes: CYP4G35 and CYP4G36.

**Figure 1:**
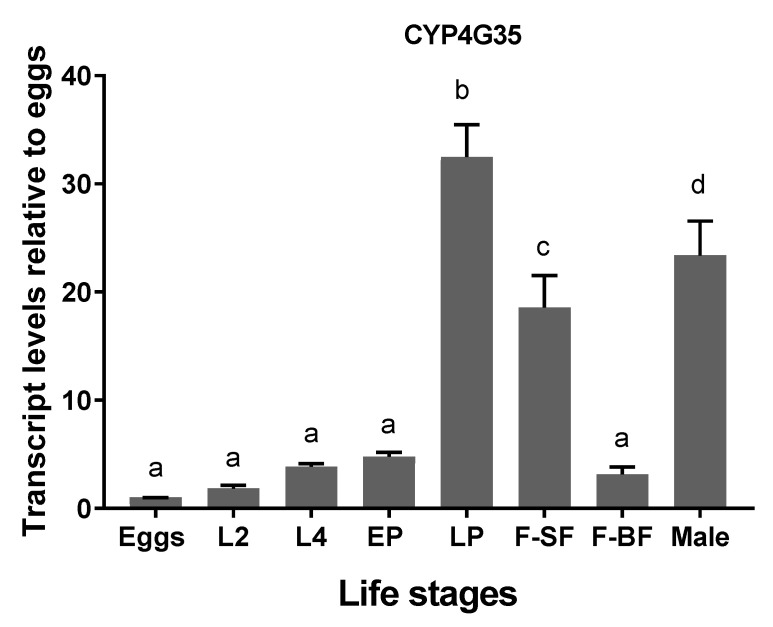
Transcript levels of CYP4G35 in *Aedes aegypti* life stages. qRT-PCR was used for relative transcript expression and 2^−ΔΔCt^ was used for analysis. Egg samples were used as a control for relative expression. The experiment was replicated three times with different biological cohorts of mosquitoes. One-way ANOVA was used with Tukey’s multiple comparison test. L2 = second instar larvae, L4 = fourth instar larvae, EP = early pupae (within 6 h of molt), LP = Late pupae (pharate adults), F-SF = sugar-fed female (24 h old), F-BF = blood-fed female (24 h post blood meal, 5 d old), Male (24 h old). *N* = 9. Mean ± SD. The letters above bars show statistical differences among samples.

CYP4G35 expression: Per their putative function, CYP4G35 expression was low in aquatic life stages; however, the late pupal stage had significantly higher expression (~35 fold higher than eggs) (Figure 1). Newly eclosed males and females also had significantly higher expression compared to eggs and other aquatic life stages but significantly lower than late pupae. Females after 24 h of blood-feeding had transcript levels similar to that of the aquatic stages (Figure 1).

dsRNA Uptake. The gene selected here highly expresses in late pupae and newly eclosed adults (Figure 1), making adult RNAi less effective. Therefore, we attempted to knock down CYP4G35 in pupae by adding dsRNA to nuclease-free DI water. Newly molted pupae were able to uptake dsRNA without any prior treatment. However, older pupae (24–36 h old) that have already melanized (dark brown to black) did not show efficient knockdown (data not shown). The number of pupae that successfully emerged as adults did not differ among CYP4G35 dsRNA, EGFP dsRNA, and water controls (Table 1).

**Figure 2:**
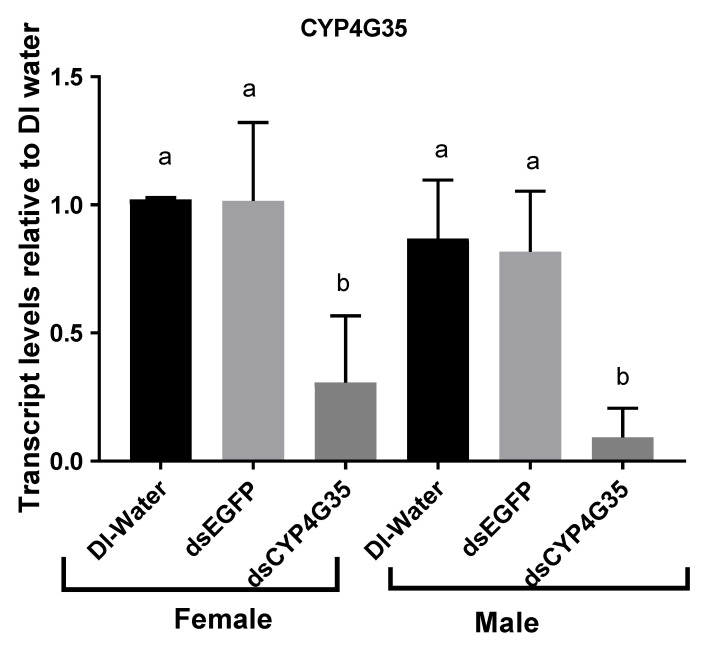
Transcript levels of CYP4G35 in *Aedes aegypti* adults soaked in dsRNA as pupae. qRT-PCR was used for relative transcript expression and 2^−ΔΔCt^ was used for analysis. Newly molted pupae were soaked in dsRNA or kept in water control until eclosed. Two-day-old adult males and females were collected for RNA extraction. The experiment was replicated three times with different biological cohorts of mosquitoes. *N* = 9. Mean ± SD. The letters above bars show statistical differences among samples.

CYP4G35 Knockdown. Expression of CYP4G35 was successfully knocked down in pupae (Figure 2). Knockdown efficiency varied in female pupae (60–99%) more than the male pupae (79–98%) (Figure 2). The knockdown persisted and expression remained suppressed in adults on day 2 after adult eclosion (Figure 2). Mortality was not significantly different in CYP4G35 and EGFP RNAi (Table 1).

Four additional genes: CYP4G36 (XM_001648326.2), insulin-like peptide 5 (ILP5; DQ845758.1/XM_001662872.2), ILP6 (DQ845755), and ILP7 (DQ845757/XM_021853884.1) were also selected for validation of our protocol. However, we only tested the protocol with one cohort (10 pupae incubated and two adults used for qRT-PCR); therefore, these data are presented in the Supplementary Materials (Figure S1, Table S1).

## 4. Discussion

RNAi by injection in adult mosquitoes works well for most genes [14,24,25]; however, it may not be useful for functional analysis of genes that are highly expressed in larval and pupal stages. Successful RNAi in *Ae. aegypti* larvae has been debatable. A few studies have shown some success with feeding dsRNA embedded in chitosan or agarose [18,26,27]. However, these methods have been limited in use. Other studies have shown successful larval RNAi by soaking mosquito larvae in water without any carrier or osmotic pressure [15,26,28]. However, no other study has evaluated RNAi by soaking mosquito pupae. Here, we show a simple RNAi method by adding dsRNA directly to water and soaking pupae in this dsRNA water. We exposed individuals right after they molt (4th instar to pupae) when the cuticle is possibly more permissive. Another study in *A. gambie* pupal RNAi by injection also suggested that the early pupal stage, before cuticle tanning was complete, resulted in optimum RNAi efficiency and pupal survival post-injection [13]. We hypothesize that the dsRNA intake is due to indirect uptake instead of feeding because pupae do not feed and still showed significant knockdown. The pupal mortality was higher in our experiments compared to Regna et al. [13]; however, the mortality was not significantly different between the control and experimental sets (Table 1). The higher mortality could be due to mechanical injury to the pupae during transfer by the paint brush. In future experiments, we will use 4–6 h old pupae to avoid injury to newly molted pupae.

This technique does not require higher concentrations of dsRNA compared to the adult injections, which further adds to the simplicity of the protocol and offers a new method for RNAi in *Ae. aegypti*. The knockdown persisted in adults at least for 2 days, which makes this method useful for many applications. We did not test knockdown efficiency beyond 2 d old mosquitoes, but in the future, it will be interesting to investigate the knockdown persistence at later time-points.

The efficiency of gene silencing is highly variable and depends on several factors including transcript and protein turnover rates as well as dsRNA uptake efficiency by tissues [24]. Therefore, not all genes are likely to respond in the same manner as we document for CYP4G35. We tested an additional four genes for pupal RNAi; another CYP4G gene, CYP4G 36 and three ILPs [29], with success rates varying from 7–80% (Supplementary Figure S1). In our hands, genes that have high expression, in general, are better suited for the knockdown by soaking. We also noted that the RNAi efficiency was much higher in younger, newly molted pupae (0–6 h old) compared to 24 h old pupae (data not shown) this is probably because of the easier penetration of dsRNA before complete melanization of pupae. Regna et al. [13] also showed better efficiency and higher survival of early pupae (lighter) compared to the late pupae (darker).

Although the utility of several delivery systems for administering dsRNA to mosquito larvae [18,27] has been successfully tested, the simplicity and ease of the method we propose may be of special interest as a tool for developing quick screens for evaluating gene function in mosquito larvae and pupae. This is the first report on RNAi in mosquito pupae by soaking and in our experience, pupae are amenable to RNAi in a similar way to larvae.

## Figures and Tables

**Table 1:** Emergence rate, sex assessment, and adult survival in control (DI water and EGFP dsRNA-treated) and CYP4G35 dsRNA-treated *Aedes aegypti*.

	DI Water	dsEGFP	dsCYP4G35
Total number of pupae treated	65	65	65
Number of pupae survived to adulthood	34	28	28
Pupal mortality (%)	41.7%	56.92%	56.92%
Total number of males eclosed	12	19	18
Total number of females eclosed	22	9	10
Total adults survived to day 2 (%)	100%	100%	100%

## Supplementary Materials

The following are available online at https://www.mdpi.com/article/10.3390/insects12070634/s1, Figure S1: Transcript levels of CYP4G36, Insulin-like peptide (ILP) 5, ILP6, and ILP7 in *Aedes aegypti* adults soaked in dsRNA as pupae, Table S1: Primer sequences and product size of genes for RNAi.
