# Supplementary material for: RNAi by Soaking Aedes aegypti Pupae in dsRNA
Source: Insects. 2021 Jul 13;12(7):634. doi: 10.3390/insects12070634 (PMC8307132; doi:10.3390/insects12070634)
Supplement: Supplementary file 1 [file insects-12-00634-s001.zip › insects-1237541-supplementary.pdf]

Supplementary Materials

RNAi by soaking *Aedes aegypti* pupae in dsRNA

Fiza Arshad, Arvind Sharma, Charleen Lu, and Monika Gulia-Nuss*

Department of Biochemistry and Molecular Biology, University of Nevada, Reno, NV, USA

Methods:

RNAi set up: As described in the main article, 500 µl of nuclease-free DI water was added to each well containing five pupae. 5µg (2µl) dsRNA was added to the DI water. Additional 1 ml of DI water was added on day 2 of treatment in the experimental and control plates. Primers are listed in Table S1.

Knockdown validation: Pupae were kept in cages for adult emergence. RNA was extracted from individual 2 D old adult female mosquitoes. cDNA was used for qRT-PCR. Ribosomal protein S7 was used for the normalization of the data. dsEGFP samples were used as a control. All primers are listed in Table S1.

Statistical Analysis: Data were analyzed using GraphPad Prism 7 software (La Jolla, CA, USA). Relative expressions were compared with one-way ANOVA, followed by Dunnet's multiple comparison test using EGFP dsRNA-treated mosquitoes as a control.

Results:

Gene selection: We selected four additional genes for the protocol validation with varied expression in pupae and adults. In mosquitoes, CYP4G subfamily has two genes. CYP4G36 in *Ae. aegypti* has a similar expression to CYP4G35 and is highly expressed in late pupae and newly eclosed adults. Insulin-like peptides (ILPs) have varied expression, ILP5 has low expression in pupae whereas ILP6 highly expresses in pupae and newly eclosed adults. ILP7 expresses only in head tissues in pupae and adults and expression is lower in pupae compared to adults [29].

Knockdown efficiency: Knockdown efficiency varied from gene to gene. In general, genes that had higher expression in the pupal stage were efficiently knockdown (56% knockdown in CYP4G36 and 81% in ILP6) whereas genes that have low expression were not significantly knockdown (26% in ILP5 and 7% in ILP7) (Fig. S1).

Supplementary Figure 1: Transcript levels of CYP4G36, Insulin-like peptide (ILP) 5, ILP6, and ILP7 in *Aedes aegypti* adults soaked in dsRNA as pupae. qRT-PCR was used for relative transcript expression and $2^{-\Delta\Delta Ct}$ was used for analysis. Newly molted pupae were soaked in dsRNA until eclosed. 2d old adult females were collected for RNA extraction. The experiment was carried out once with 10 pupae from the same biological cohorts of mosquitoes. Two adult females per treatment were used individually for expression analysis. EGFP dsRNA treated mosquitoes were used as a control for relative expression. N=2. Mean ±SD. **P value=0.004; ***P value=0.0004

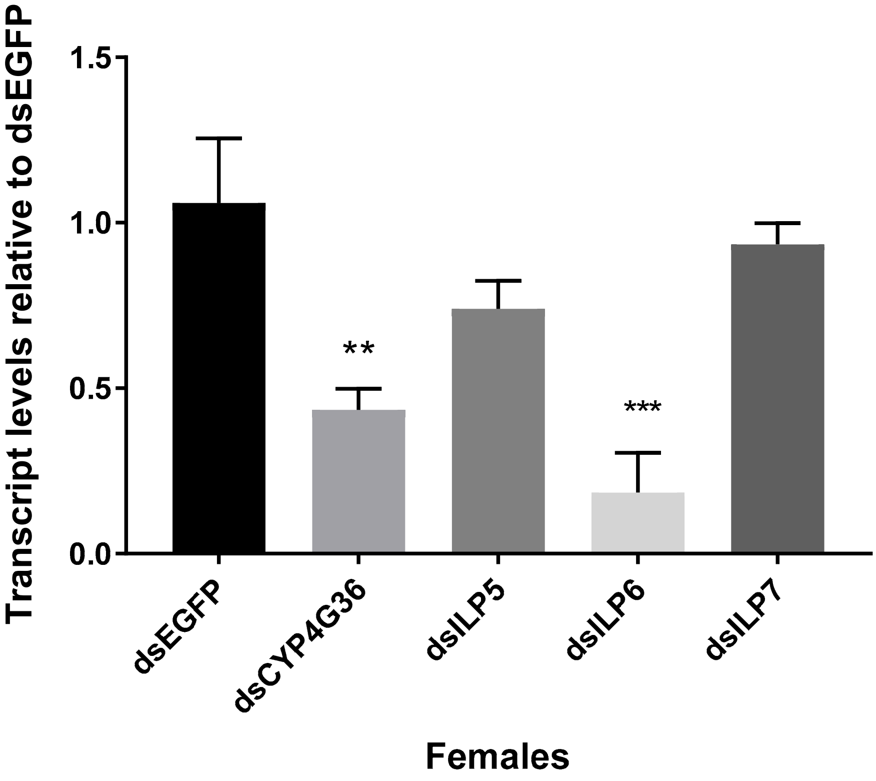

Supplementary Table 1: Primer sequences and product size of genes for RNAi.

Gene name	Forward Primer Sequence	Reverse Primer Sequence	Product size (bp)
AaCYP4G36	5’CGCGTCATCTCAGACCTCAA3’	5’AGGTTTTTGGTGTCCCGGAG3’	212
T7AaCYP4G36	5’TAATACGACTCACTATAGGGAGAAAAATGTCCGCGACGGTTG3’	5’TAATACGACTCACTATAGGGAGATTTCCACGGTACACTCGCTC3’	595
AaILP5	5’GTTGGCGGCTGACTATCC3’	5’CCGGTAAGAAACCACATACG3’	148
T7AaILP5	5’TAATACGACTCACTATAGGGTGGTGAACTCGCCAAGTGTT3’	5’TAATACGACTCACTATAGGGAAAGCTTTGCGCTTGCTCT3’	481
AaILP6	5’GAGCAAATCCACAACTCCAG3’	5’GCACAGTTCCAAATTCCATC3’	104
T7AaILP6	5’TAATACGACTCACTATAGGGAGATTGTCGTTTTCCTTCTAATA3’	5’TAATACGACTCACTATAGGGAGATAGTAATGTTTTCGTCTG3’	446
AaILP7	GACAACGCCAATAAACTGCC	CGGTGAGTGTATCCGTAAGC	163
T7AaILP7	5’TAATACGACTCACTATAGGGCAAGCAAGCGTCGAGTGATG3’	5’TAATACGACTCACTATAGGGTCACATTTAGACACAGCACTATCAA 3’	405
